# Eritrean Refugees’ and Asylum-Seekers’ Attitude towards and Access to Oral Healthcare in Heidelberg, Germany: A Qualitative Study

**DOI:** 10.3390/ijerph182111559

**Published:** 2021-11-03

**Authors:** Yonas Semere Kidane, Sandra Ziegler, Verena Keck, Janine Benson-Martin, Albrecht Jahn, Temesghen Gebresilassie, Claudia Beiersmann

**Affiliations:** 1 Heidelberg Institute of Global Health, Heidelberg University Hospital, 69120 Heidelberg, Germany; yonas.kidane@alumni.uni-heidelberg.de (Y.S.K.); verena.keck@t-online.de (V.K.); janinebensonmartin@gmail.com (J.B.-M.); temesgenbk@gmail.com (T.G.); beiersmann@uni-heidelberg.de (C.B.); 2 Department of General Practice and Health Services Research, Section Health Equity Studies & Migration, Heidelberg University Hospital, 69120 Heidelberg, Germany; Sandra.Ziegler@med.uni-heidelberg.de (S.Z.); 3 Gesundheitsamt Enzkreis, The Public Health Office Enzkreis, 75177 Pforzheim, Germany

**Keywords:** oral health care, dental, access, attitude, Eritrea, refugees, asylum-seekers, qualitative

## Abstract

Oral health concerns in Eritrean refugees have been an overlooked subject. This qualitative study explored the access of Eritrean refugees and asylum-seekers (ERNRAS) to oral health care services in Heidelberg, Germany, as well as their perceptions and attitudes towards oral health care. It involved 25 participants. We employed online semi-structured interviews (*n* = 15) and focus group discussions (*n* = 2). The data was recorded, transcribed, and analysed, using thematic analysis. The study found out that most of the participants have a relatively realistic perception and understanding of oral health. However, they have poor dental care practices, whilst a few have certain misconceptions of the conventional oral hygiene tools. Along with the majority’s concerns regarding psychosocial attributes of poor oral health, some participants are routinely consuming Berbere (a traditional spice-blended pepper) to prevent bad breath. Structural or supply-side barriers to oral healthcare services included: communication hurdles; difficulty in identifying and navigating the German health system; gaps in transculturally, professionally, and communicationally competent oral health professionals; cost of dental treatment; entitlement issues (asylum-seekers); and appointment mechanisms. Individual or demand-side barriers comprised: lack of self-sufficiency; issue related to dental care beliefs, trust, and expectation from dentists; negligence and lack of adherence to dental treatment follow-up; and fear or apprehension of dental treatment. To address the oral health burdens of ERNRAS, it is advised to consider oral health education, language-specific, inclusive, and culturally and professionally appropriate healthcare services.

## 1. Introduction

To date, literature regarding the magnitude of oral health burdens of the widely dispersed Eritrean refugees and asylum seekers is scarce. The world is experiencing a surging number of forcefully displaced persons with 70.8 million in 2019. They were either refugee (25.9 million), internally-displaced persons (41.3 million), or asylum-seekers (3.6 million) [1]. The UN High Commissioner for Refugees (UNHCR) defined a refugee as “someone who is unable or unwilling to return to their country of origin owing to a well-founded fear of being persecuted for reasons of race, religion, nationality, membership of a particular social group, or political opinion [2]”. An asylum-seeker, though, is “someone whose claim has not yet been finally decided on by the country in which the claim is submitted [3]”. By the end of 2018, 10% of the world’s refugees resided in Europe [4] with Germany hosting the largest number [5]. Eritrea, despite its small population of an estimated 3.5 million in 2018 [6], is ranked ninth as a country of origin of refugees with almost 15% of its population living in the diaspora [5]. By the end of 2018, there were 55,300 Eritrean refugees in Germany [1] and Eritreans were ninth by nationality of all applicants seeking protection in Germany [7].

Even as many refugees were able to escape from threats of persecution; many often failed to avoid the risks associated with poor health conditions. There is evidence to suggest that the general health of refugees is inferior in comparison to that of the host population [8,9,10]. Correspondingly, the oral health of refugees and asylum-seekers was poor in comparison to that of the general host country’s population [11,12,13,14]. Though data is scarce, it was found that refugees and asylum seekers have a higher prevalence of oral disease and lower oral health status than their counterpart native Germans [15,16].

According to the World Health Organization (WHO), oral disease burdens are one of the leading health problems that refugees experience [17]. Among refugees, there is a high prevalence of major oral diseases such as dental caries, periodontal disease, malocclusion, missing and fractured teeth, orofacial trauma, and orofacial malignancies [11,13,18,19,20,21]. A study among newly-arrived refugees in Massachusetts indicated that oral diseases were the most common complaint in children and the second most common in adults [22]. Another study in Brussels, Belgium, also showed that dental conditions were the second most frequent diagnosis following respiratory tract infections [23]. This suggests that dental care is suggested as a pressing healthcare need of many refugees and asylum seekers [14,24,25].

Among the principal factors contributing to poor oral health are: ill-equipped or inaccessible dental healthcare in their Country of Origin (COO); lack of dental care in migration transit or refugee camps; and poor personal or cultural dental care practice [24,26,27,28]. While many appear to have reasonable perceptions and understanding regarding the significance of good oral health as holistic health [28], their overall oral health knowledge, attitudes, and good practice remain unsatisfactory [29,30,31]. Literature indicates, however, that the principal source of poor oral health status among refugees is, more often than not, actually related to limited access to dental care in the host country [24,26,28]. The German legislation is restrictive. For example, in Germany, access to dental care services according to *‘§4 and § 6 Asylum-Seekers’ Benefits Act (AsylbLG)* is limited during the first 18 months after arrival [32]. In essence, the legislation severely limits access to general and oral health care [32]. Other barriers described as limitations to care in the host country include language and communication issues; fear of dental treatment; anxiety and trust issues; high treatment costs; low income; distance to a dental clinic; quality of care; restricted treatment choice; long waiting lists and time; low oral health literacy; and other cultural and psychological barriers [12,28,30,33,34,35].

According to the WHO, oral health is a crucial indicator of overall health, wellbeing, and quality of life [36]. Many of the oral conditions, comprising periodontitis, tooth loss, dental caries, and oropharyngeal infections, share modifiable risk factors (high-sugar diets, poor oral hygiene and care, and excessive alcohol and tobacco use) with the leading non-communicable diseases including diabetes, cancer, and cardiovascular and respiratory diseases [37,38,39,40]. When the connection of oral health to quality of life was measured [36], the Oral Health-Related Quality of Life (OHRQoL) of refugees was found to be very low [41,42]. Despite this knowledge, oral health care is frequently neglected and undervalued as a vital healthcare service to refugees [43]. The UNHCR has yet to assign it within the significant health framework for the refugee population [44]. To date, Canada remains the only country ever to develop specific guidelines for oral health screening of refugees and immigrants [45]. While refugees’ oral health care remains a pressing issue, studies are scarce [24,30]. In addition, almost all of the studies concluded that oral health is less understood and above all less accessible to these disadvantaged populations [15,24,28,29].

Thus far, no qualitative research has been published in Germany looking at the refugees’ perspectives, understanding, experience, and the main difficulties associated with oral healthcare access and utilization. In particular, no study has focused on these aspects of oral health among Eritrean refugees in Europe or in Germany.

Therefore, this study aims to close this research gap. It explores the access of Eritrean refugees and asylum-seekers (ERNRAS) to oral health care services in Heidelberg, Germany, as well as their perceptions and attitudes towards oral health care.

## 2. Materials and Methods

This study was conducted using the qualitative research method. As this study seeks to understand an individual’s experiences regardless of any preconceived ideas [46], as such, when describing and interpreting the data, participants’ own perceptions, understandings, and perspectives were taken into account.

### 2.1. Study Setting

The study took place in the city of Heidelberg, Baden-Württemberg, Germany. Due to the influx of refugees in 2015 and Baden-Württemberg being the second largest state, it received a high number of asylum applications for three consecutive years (2016–2018) [47,48]. On the outskirts of the city of Heidelberg, the Patrick Henry Village (PHV), a former US army housing area, is used as a refugee arrival and registration centre [49]. Heidelberg city itself is the home to more than 450 refugees and asylum seekers, including many ERNRAS [50].

### 2.2. Sampling Procedures

Participants were selected through exponential non-discriminative snowball sampling, in which the first participant recruited provides multiple referrals. Each new reference offers further information for referral until sufficient participants are enlisted [51]. As the principal researcher (Y.S.K.) is Eritrean, he was able to socialise and exchange addresses with potential participants before the COVID-19 pandemic. He visited places where Eritreans usually gather such as Eritrean restaurants, and Eritrean church services in Heidelberg. After a series of snowballing and referrals, the first author had invited 31 ERNRAS to participate in the study of which 25 finally participated.

Of these, 84%, were refugees (individuals granted a refugee status), and 16% were asylum seekers. On average, participants had lived in Germany for 4.4 years (range: 2–7 years), and the majority, 76%, have lived there for more than three years. The study sample consisted mostly of men, 76%; the mean age of participants was 29 years (Range: 19–52); with more than half having a secondary level education. The majority of participants (60%) were employed at the time of the interview. Most of them, 72%, are unmarried. The sample characteristics are shown in Table 1.

### 2.3. Data Collection Instrument and Procedures

Data collection took place in April and May 2020. The principal researcher (Y.S.K.) carried out fifteen individual in-depth interviews (IDI) and two focus group discussions (FGDs) (with 6 participants each) in Tigrinya (Eritrean official language). The data collection tools consisted of a semi-structured in-depth interview (IDI), as well as a focus group discussion (FGD) guide. These guides were developed by the principal researcher with assistance from the co-authors (C.B., J.B.M.). A pilot test was performed among three Eritreans (friends and colleagues of Y.K.S.) before final adoption. The guides covered a wide range of topics within the following subject areas: perception of oral healthcare, understanding of oral health determinants, dental care behaviour, and barriers of access to oral healthcare services (see Appendix A, Table A1).

Data collection was originally planned face-to-face, however was conducted online via video conference software (Skype or WhatsApp) because of the COVID-19 pandemic restrictions. Each interview lasted an average of 40 min, with the FGDs lasting one hour and fifteen minutes. The FGDs were protocolled by an assistant (T.G.). The conversations were audio-recorded in Tigrinya, transcribed in Tigrinya, and translated into English for analysis by the first author (Y.S.K.).

### 2.4. Data Analysis

In order to organize, process, and manage the data, NVivo 12 (QSR International, Melbourne, Australia), a qualitative data analysis programme, was used. Data were analysed using thematic content analysis. This method supports identifying, analysing, and interpreting patterns of meanings (themes) within qualitative data [52]. A framework developed by Levesque and colleagues in 2013 [53], comprehensively addresses access to health care. The framework was used to code deductively for predefined themes in the data. According to the framework, healthcare accessibility involves five supply-side dimensions (provider-side): Approachability, Availability and Accommodation, Acceptability, Affordability, and Appropriateness. Correspondingly, there are also five dimensions (conceptualized as abilities) paralleling on the demand-side (user-side): the Ability to perceive, the Ability to seek, the Ability to reach, the Ability to pay, and the Ability to engage [53]. In addition to this deductive coding, the researcher read, explored, and coded the dataset for patterns and themes that emerged inductively.

**Figure 1:**
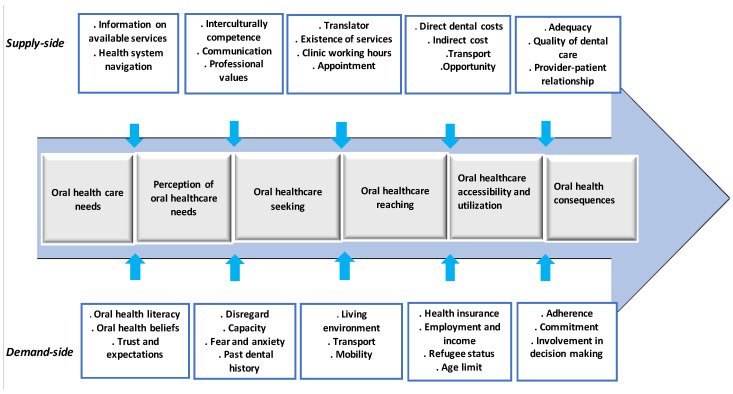
Conceptualization of oral healthcare access among ERNRAS in Heidelberg, Germany, adopted from the access framework of Levesque et al. (2013) [53].

Finally, eight major themes could be identified—three themes, mainly on the perception of oral healthcare, understanding of oral health determinants, and dental care behaviour of ERNRAS. The five subsequent themes depicted are in line with the five principal dimensions of access to healthcare and their equivalent users’ abilities adopted from the aforementioned conceptual framework developed by Lévesque et al. (see Figure 1).

### 2.5. Ethical Consideration

This study was conducted in accordance with the Declaration of Helsinki, and the protocol was approved by the Ethical Commission of the Medical Faculty of Heidelberg, Germany (protocol number: S-207/2020). Study participation was voluntary, and all had given their written informed consent. Confidentiality was protected by the use of pseudonyms when storing, analysing, and reporting the data. No incentives or compensation was given to the participants.

## 3. Results

The findings of this study are presented according to eight emerging themes and sub-themes. For a detailed overview please see Appendix B, Table A2.

### 3.1. Perception of Oral Health Care

In exploring the perception and perspective of Eritrean refugees and asylum seekers, good oral health was described as having white teeth, a pleasing smile without any decayed, broken or crooked teeth, and also no gum bleeding or bad breath: *“I would say, we shouldn’t have a dental cavity, bleeding gums, bad mouth odour, or neither broken nor crooked tooth” (FGD-1).* They further commented on the vital significance of oral hygiene as part of personal hygiene and the valuable benefit of regular oral healthcare in boosting self-esteem and social approval. One of the respondents remarked: “*Earlier, I lost one of my front teeth from a fall injury; it was so horrible to see myself in front of a mirror (laughter) […]. I used to feel so embarrassed in public and I used to cover my mouth all the time. It was so awful to see your tooth missing” (FGD-1).* Commenting on social uneasiness and suffering from bad breath, some participants also cited and believed that eating a traditionally prepared spice-blended pepper (Berbere) neutralises bad breath. Berbere is a traditional Eritrean spice blended pepper powder, mainly containing chilies, garlic, fenugreek—it is an ingredient in most Eritrean dishes:

*“I believe Berbere protects you from bad mouth odour! As for me, I am getting Berbere from home [Eritrea] solely prepared by my mother, and I am consuming it every day. You know what […]? as Berbere is my routine food, I do not have any terribly smelling mouth like others do” (IDI-13)*.

### 3.2. Understanding of oral Health Determinants

The study sought participants’ views on the determinants of oral healthcare. The majority noted the close association between oral health and general health and remarked critically on the seriousness of oral diseases. One member of the discussion said: *“As molar tooth pain can go the head […], it makes it so dangerous and risky to your life. And from what I heard and also experienced […], there is no other pain cause more anguish than dental pain” (FGD-2)*. Most of the participants believed that the risk factor for their poor oral health was related to their lifestyle: poor oral hygiene routine, sweet and starchy food consumption, trauma, and tobacco smoking. The majority, however, have stressed that the dietary transition from a fibrous and low-sugar traditional diet back in Eritrea to a high-sugar or processed food in Germany as the major risk factor:

*“Life in Europe is somehow different from our country. Most of us here [in Germany] we tend to change our lifestyle. We start to eat differently, like sweet and packaged food that are not common in our country; starting from me, smoking isn’t also uncommon. I believe that those things are the reason for my poor oral health” (IDI-4)*.

### 3.3. Dental Care Behaviour

As we enquired about personal and professional dental care practices, most of the respondents acknowledged the fundamental function of routine oral cleanness in preventing and reducing dental diseases. Almost all mentioned exercised some form of oral hygiene routines that varied from once, 6 (24%), or twice, 16 (64%), a day to an irregular basis, 3 (12%), using toothbrushes and toothpaste. In addition, eight (32%), also spoke about their habit of mouth washing in addition to toothbrushing or separately. However, the majority are either not using, or unaware of, dental flossing as a complementary oral hygiene method: “*I have no comments on this method of cleaning teeth. […] honestly, I know nothing and have also never used it” (FGD-2).*

Few participants, five (20%), have been using tooth twigs (Mewets), a traditional Eritrean teeth cleaning tool, similar to Miswak (Asia, Africa, and the Middle East) [54], which is prepared mainly from two tree branches, those of the Olive tree (Olea Europea subspecies. Africana) and of the Sand olive (Dononaea Angustifolia). The dimensions are 6–10 cm long and 4–10 mm thick. The stick is applied to the teeth to scrub the surface in a horizontal or vertical motion until the twig split thereby allowing one to clean between the teeth as well as massaging and cleaning the gums [55]. When asked about their perception of using Mewets in Germany, their responses were mixed, as most of them acknowledged their habit of applying Mewets as the only tooth cleaning tool in Eritrea but had now changed to other methods: *“I have never used the twig in this country [Germany]; I couldn’t find the right tree. I don’t have any choice but to use the toothbrush” (IDI-6).*

Regarding the frequency of dental attendance, only, two (8%), participants cited visiting the dentist regularly and diligently on a bi-annual basis while two (8%) admitted that they have never attended a dental clinic in their lifetime. The majority’s main reason for a dental visit was as a result of dental emergencies: *“The only time I went to my dentist was [..], the day that I experienced very serious dental pain” (FGD-2).*

Some participants raised doubts over their current regular oral hygiene tools and materials. Two (8%), participants of the FGD commented negatively regarding the regular use of toothbrush and paste: *“If we use the toothbrush frequently, with toothpaste after every meal, I believe that it may damage our tooth” (FGD-1).* One female participant also assumed that the regular utilization of a dental toothbrush widened the gaps between her teeth. As well, irregular, or intermittent use of a toothbrush was considered as a risk factor for bad breath by another participant: “*If we habitually brush our teeth and stop, we may expose ourselves to bad mouth odour” (IDI-13).* Most of the participants also voiced their concerns regarding dental flossing: *“I know about the thread […], I believe, if you keep on doing it, you can harm your gums now and then” (FGD-1)*.

### 3.4. Approachability and Ability to Perceive

This theme refers to the capacity of refugees or asylum-seekers to discover dental care services and the availability of adequate oral health information sources that influence an individual’s judgment of access to dental care facilities [53]. Obtaining clear information, locating dental services, and navigating the German health system, was found to be a complex and inconsistent endeavour for most of the ERNRAS. Many of the newly arrived ERNRAS remarked on the challenges associated with finding reliable information on health services, or a person to guide them through the health system. A recently-arrived mother of three, asylum-seeker, commented: *“No one would show or take you to a dental clinic. You have to find it on your own; and it was so difficult to understand and to find out where the dental clinics are” (IDI-2).* The majority of the respondents also reported on their difficulties of navigating the health system in Germany in general: “*You have no idea […]! it is so challenging to understand how the health system works. There is limited or no information about where, how, and when to approach the eye clinic, the dental clinic and so on” (IDI-6)*.

Although the majority of the participants believed that they have basic oral healthcare literacy, few said anything about how far their lack of exposure to proper professional dental care in Eritrea, had impacted their overall oral healthcare mentality in Germany. They also expressed their strong beliefs in traditional medicine such as potions, herbs, or prayers, as influences on their oral healthcare perception:

*“Back in our country [Eritrea], if we experience any kind of illness, we don’t simply go to the clinic […]. Our parents and community healers used to give us any traditional herbs, potions, and spells. Then we wait for God to heal us. Likewise, here [in Germany] even though I am not using the herbs and potions […], I simply don’t go to the clinic, I just pray at home and wait for God to heal me from my misery” (FGD-1).*

Most participants spoke highly of and trusted their dentists: *“My dentist is so reliable and honest […]. She is always helpful and she treated almost all of the dental problems I had” (IDI-2).* Some participants, however, disagreed with their health providers’ treatment decisions, as well as the bureaucracy involved in dental healthcare for ERNRAS in Germany. They also reported their concerns about the unforeseen forthcoming financial burden associated with dental health care:

*“Sometimes though, the dentists work on a tooth that you have not complained about and we might not be comfortable with it too. As far as I am concerned, I don’t like it” (IDI-13).*

*“For some of us, it is like we don’t even trust some of the dentists in Germany. I think that when they [dentists] are taking out our teeth, they want to do so in their own interest, and to replace ours with artificial teeth, which is not in our interest” (FGD-1)*.

*“I don’t trust the dentists too. I have a trust issue! I mean […], the bureaucracy is very tedious […], they tell you to sign here, and there […], I don’t know what we are sometimes saying. Who knows, later they [dentists] might ask us to pay all (laughter)?” (FGD-1)*.

### 3.5. Acceptability and Ability to Seek

This theme conveys the intercultural and social competencies of oral healthcare providers to accept refugees, and the ability of refugees to seek dental care services [53]. In addressing that, some participants mentioned a lack of interculturally proficient dental care professionals. One participant iterated:

*“The dentists should try to understand our difficulties in learning the new culture here [Germany]. In our country [Eritrea], we have a different background and practice for tooth care. We don’t know much about the new way of dental care in Germany […], but we used to treat dental pain with herbs. Thus, the doctors should show some kindness and teach us calmly the correct way […]. My dentist expects me to comply to whatever he said, and he is very rigid and strict […]. I really didn’t understand his instructions and he once yelled at me too (sigh)” (FGD-1)*.

The majority of the participants are satisfied with the services that they obtain at the dental clinics: *“As my former dentist is so cooperative, I also take many of my fellow Eritreans, who don’t understand about their dental health, to her and get the treatment” (IDI-1).* A few, however, have commented on communication and conduct issues of some dental professionals: *“My former dentist had a very arrogant receptionist. I wasn’t really comfortable with her. I was discouraged from going to the clinic as I couldn’t stand her discriminating look; I take only pain killers and stay at home” (IDI-2)*.

Some participants reported a great deal of uncertainty in their capacity to seek dental care. They mentioned that they either were not confident or not independent: “*I once wanted to visit a dental clinic but I couldn’t. I honestly had no enough confidence to talk about my complaint” (IDI-6)*. Furthermore, despite understanding the need for regular dental visits, some participants admitted to negligence or indifference. They believed that this was deeply rooted because their upbringing in Eritrea most often did not emphasise the significance of regular dental check-ups and care. One participant also alluded to the widespread and serious suffering on his migration journey (Sahara-Libya-Mediterranean Sea) and using this to relativise and justify his non-use of dental care:

*“As far as I am concerned, the reason behind my hesitation in visiting a dental clinic, despite experiencing marked dental problems, is that I had been through a very bad experience on my way to Europe. I saw and witnessed a lot of awful distress and health problems along my way in Sahara, Libya or at sea [Mediterranean]. Comparing to those, I consider my teeth problem as a simple discomfort and I just resist the pain until it resolves itself” (FGD-1)*.

The majority of the participants explained why they opt out of regular dental visits. They usually related this with the presumptive or experienced fear and apprehension of dental instruments or physical dental pain.

*“I chose not to go back after six months because I hate the machines that trim the teeth. Do you know how annoying are the rotating machines and the other sharp instruments that they [dentists] use? For example, one day, I had experienced a severe headache because of the instruments that they had stuck into my teeth; honestly, I hated it. Now that I am treated, thanks God it’s over […]. It has been three years since I have experienced any kind of dental problem, and I never been in a dental surgery after that too” (IDI-15)*.

### 3.6. Availability and Accommodation and Ability to Reach

This theme relates to the availability of services that enable refugees or asylum-seekers to access dental care, as well as their abilities to reach the dental care facilities [53]. Communication is found to hamper ERNRAS access to oral healthcare. This was either a result of a language barrier or the non-availability of a translator. Participants confirmed that language problems were the most significant challenge in accessing dental care or support: *“It is the language problem; I can’t tell a dentist what is really happening to me, and that is why I didn’t go to them [dentists]” (IDI-6).* In addition, nearly all of the participants were concerned about the unavailability of interpreter service and believed that visiting a dentist without a translator could be a source of both misinformation and non-compliance with instruction: *“For example, I had severe dental pain, and I was waiting for artificial teeth. I had to go for several successive appointments, and I asked for a translator, but they [dental team] couldn’t find me one. Thus, I missed several instructions from the dentists” (IDI-3).* Furthermore, dependency on an interpreter and the issue of privacy and confidentiality was also mentioned as a barrier by some participants: *“I might* *find a translator who can help me translate, but I also don’t want to share my health problems with people of my own community as he or she might publicise my health issues” (IDI-14).*

The majority of ERNRAS expressed satisfaction with dental services in Germany. However, some were discontented over rigid clinic working hours, long waiting lists or times, inflexibility of dental appointments, and the long-distances or mobility issues as a hindrance to access to dental care:

*“Most of the appointments that you get are on weekdays […], where most of us are busy at work or school […]. They [dentists] won’t see you at weekends. So, if we need further visits, we couldn’t miss work or classes so often […]. Thus, we often miss follow-up appointments” (FGD-1)*.

*“I can say that there is some problem, especially for those who reside in villages, where train transport is unpredictable […]. Pregnant mothers have some access problems. My friend’s wife was once caught up in such a difficulty” (FGD-1)*.

### 3.7. Affordability and Ability to Pay

This theme describes the financial ability of refugees or asylum seekers to devote enough funds and time to expend on dental care services and their ability to generate capital to finance the services [53]. Although some participants mentioned the free-of-fee primary dental care services, which are covered by insurance, the majority, however, made it clear that cost is a significant impediment to obtaining dental services. They reported that most of the dental treatments except regular check-ups, teeth cleaning, and tooth filling, are out-of-pocket or require co-payment. Indirect costs like those of transport, dental products, and opportunity costs were also mentioned by some participants as a detrimental factor in accessing dental services:

*“In my opinion, comparing with the other services, dental care is expensive, and it always requires several consecutive appointments so that you need to skip work, pay for trains, and dental products like tooth brush, paste or mouthwash” (IDI-1)*.

Participants acknowledge the complexity of health insurance eligibility and entitlement procedures, i.e., how, where, and when to approach or access dental care services. Many participants experienced that eligibility for free dental services depended on factors such as age and refugee status (asylum application decisions). They also remarked on the impact of employment status when seeking dental care:

*“I haven’t had enough money to get the treatment [orthodontic treatment], because I have no work or income” (IDI-4)*.

### 3.8. Appropriateness and Ability to Engage

Here the compatibility of the dental service with the needs of refugees and their involvement in decision-making and treatment decisions were explored [53]. Most participants were content with their dentists’ diagnosis, management, and communication competence: *“My doctor is so good and tells you everything about your oral health. She effectively treated all my dental problems and also cleaned my teeth” (IDI-2)*. Some, however, have perceived, and complained about, the technical and interpersonal inadequacy and incompetence of the service providers: *“I can say that my former dentist could have done more […]. Not only did he treat my complaint badly, but he also forced me to go along with his decisions. That’s why I always complain about him, the treatments that he gave me were neither appropriate nor satisfying” (IDI-3).*

Some participants indicated that some of their providers (dentists) were not only uncooperative and bad-tempered, but also difficult to build relationships with. One participant complained about a significant amount of money that she was forced to pay for unsuccessful treatment: *“I couldn’t express my feelings. I wanted to sue the dentist […], but I don’t know how everything works, and I don’t know the court and how to take people to court too. I was so frustrated and depressed (sigh)” (IDI-12)*. Many participants though admitted to their own lack of adherence to regular dental visits and appointments: *“I have never been in the dental clinic for the last two years, after I had received a dental treatment that actually relieved me from the pain that I had. To speak from experience […], once my dentist strongly advised me to visit a dentist every six months. She told me that I am entitled to two check-ups a year and teeth cleaning [i.e., scaling and polishing]. Still, I am not adhering to her advices (laughter)” (FGD-2).*

When participants were probed about their role in decision-making regarding their treatment options, many of them reported having limited enthusiasm to engage in their treatment decisions. This, they believe, affected their motivation to become involved in dental care and commit to finishing their treatment:

*“My dentist once informed me that my tooth was decayed and suggested to extract it, and I simply agreed. Then when he [the dentist] attempted the extraction, it took him six hours. Since the tooth was decayed only on the upper part not at root, I should have asked him to restore it. It was my mistake. I was looking for a temporary solution but it cost me a lot and my left cheek was really numb for the following six months” (IDI-3)*.

**Figure 2:**
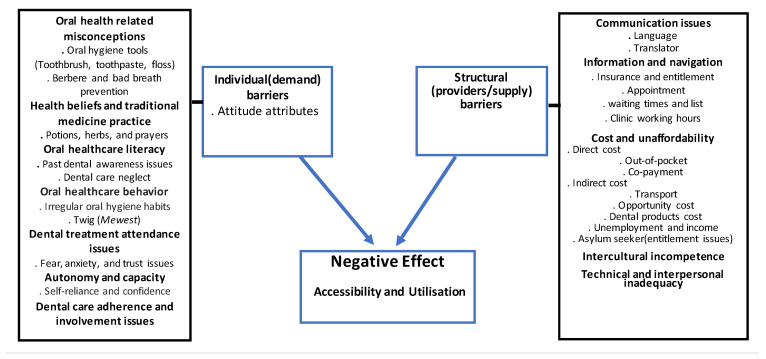
The effect of individual and structural barriers on oral healthcare accessibility and utilization of ERNRAS.

As results have been summarized in Figure 2, the oral healthcare attitude attributes of Eritrean refugees and asylum-seekers not only make proper self and dentist dental care difficult but also appear to negatively affect directly or indirectly the accessibility and utilisation of oral healthcare services as much as the supply (structural) side barriers do.

## 4. Discussion

This qualitative research identifies the major oral health concerns and barriers to dental care services among Eritrean refugees and asylum-seekers living in Heidelberg, Germany. In addressing those concerns, the results of our study indicate that the participants defined good oral health as the absence of any condition that involves problems with teeth, gums, jaws, cheeks, lips, or breath. This finding is consistent with a qualitative study carried out among newly arrived refugees in Canada, where the assessment of participants on what represents good oral health comprised absence of swelling, missing, broken, decayed or painful teeth [56]. As it has been documented in several studies among African refugees [28,57,58], ERNRAS also acknowledged the physical, mental, and social benefits of regular oral healthcare. The WHO pointed out that the oral cavity is not an isolated organ, and consequences of poor oral health are not only limited to the teeth but also affect general health [36]. In line with that report, the current study revealed that most of the participants were conscious of good oral health as part of holistic health, including the need to avoid the possible life-threatening consequences of untreated oral diseases. Compared to findings reached by Keboa and colleagues [28], our study identified the psychosocial concerns of bad breath and further new insight into a custom followed by some ERNRAS, mainly to prevent oral malodour by routine consumption of a traditional pepper (Berbere). The metal chelating activity (to stabilize and remove harmful metals), antioxidant properties, and enhancing effect on the carbohydrate-hydrolysing enzyme (hypoglycemic activity) of spice blend Berbere has been scientifically established [59]. However, the anti-halitosis element perceived and hypothesized by ERNRAS is yet to be researched.

When refugees are settling in Western countries, they are introduced to a higher-sugar diet than they were accustomed to in their COO [60]. Our findings confirm this dietary transition into the consumption of readily available ‘carb-heavy’ foods (concentrated with sugar and fat) in Germany and are perceived as a leading cause of their dental diseases. The nutritional transition and its negative impacts on the overall oral health of refugees and asylum seekers had been identified in several studies [15,29,30,61].

Dental care is the preservation of a healthy oral cavity, and it relates to regular personal and professional oral healthcare [36]. However, our study found that the majority of participants do not comply regularly with all the recommended dental care practices [15,24,28,30,61,62]. Almost all of the participants accepted toothbrushes and mouthwash, while none of them acknowledged dental floss as a significant oral hygiene tool. This result builds on the existing evidence, where a study reported that refugees were barely utilizing dental floss as a complementary device to clean their interdental areas [15]. In addition, unlike in Germany, most ERNRAS disclosed their utilisation of twig (Mewets) as a sole oral hygiene tool when they were in Eritrea. A study in Eritrea has shown the antimicrobial and anti-cariogenic (caries prevention) effect of Mewets [63]. The study might suggest the change in practice (from Mewets to a toothbrush) reflects the belief or attitude change. However, considering findings in similar studies of East African refugees [29,61], a more plausible explanation is the inconvenience and unavailability of the right tree in Germany. Regarding reasons for dental attendance, the results demonstrate that the majority only seek treatment in times of dental emergency or pain. This finding mirrors several studies amongst East African refugees living in the U.S.A [61], Australia [30], and Canada [28].

Misperception related to oral hygiene tools and methods among many East African refugees has been highlighted in previous studies [29,61]. Nevertheless, the findings from the current study go beyond previous reports, showing that some ERNRAS have negative perceptions about regular use of a toothbrush, toothpaste, and dental floss. However, they also believed that discontinuation of the routing toothbrushing could lead to halitosis (bad breath). The plausible explanation of this erroneous assumption is that users were unable to notice their halitosis before they started using toothpaste; they might not have known that an oral malodour already existed. Since our sense of smell is a learned behaviour [64], they start to differentiate bad breath from normal breath following the discontinuation or skipping of regular toothbrushing. Revisiting the belief that some ERNRAS rely on the hypothetical concept of Berbere’s advantage in preventing bad breath, brushing is not only replaced as an important means to prevent oral malodour, but also considered to be detrimental to oral health.

This study found that ERNRAS had or have difficulty in obtaining information, locating oral healthcare services, and navigating the health system in Germany. A similar conclusion was reached on recently-arrived refugees in Finland [25], and East African refugees in Australia [58]. As well, participants with limited oral health literacy and undesirable health beliefs might also hamper their dental care-seeking behaviour as a study conducted in Eritrea by Andegiorgish and colleagues shows [65]. Furthermore, since stress and psychological insecurity are endemic among refugees [13,26], a dentist and trust-based dental treatment seem to be largely unachievable [58]. Consistent with previous findings [26,57], fear of dental pain, anxiety, or past negative dental experiences of oneself, friends, or family members influenced the care-seeking behaviour of ERNRAS. Additionally, the current study goes beyond that some ERNRAS present with certain trust issues towards provider’s possible dental malpractice and unpredicted future financial implications of the current dental treatments.

The reported lack of interculturally competent professionals was a significant finding in this study. The perceived experienced discrimination, lack of empathy, and misconduct were also identified in related studies [58,66]. This underlines the importance of a dentist who understands and accepts his or her patient’s diverse cultural beliefs and background. Furthermore, in line with similar research [67,68], this study found that apart from personal dental care disregard and negligence, reduced autonomy and self-reliance are also some of the participants’ hurdles for seeking dental care.

This study suggests that communication features, language barriers, or unavailability of translators, negatively affect participants’ accessibility. This finding mirrors other studies in Europe [8,69], and in Germany [70]. It is understood to be a major barrier to seeking oral healthcare services for the majority of ERNRAS. Moreover, our findings confirm that the patent’s dependence on translators not only interferes with an effective conversation, diagnosis, and follow-up but also raises concerns on confidentially and privacy [71,72].

This study established that ERNRAS faces difficulties in understanding the working hours and appointment procedures of dental clinics. This correlates reasonably well with a study conducted by Mattila et al. [25], and further supports existing findings on the challenges of refugees’ to secure and attend dental appointments, cope with long waiting times, and make the right treatment choices [30,58,67].

Many of the participants of this study report either postponing or avoiding dental treatments because of direct or indirect costs (transport, dental products, and opportunity costs). Evidence from other studies in Germany (direct payment) [16], Australia (co-payment and indirect payment) [67], and North America (direct and indirect payment) [28], all suggested that financial difficulties discourage refugees from seeking dental treatment. Analogously, a lack of health insurance and entitlement, as well as unemployment seems to pose challenges for asylum-seekers whose legal asylum status were still being processed. Age and refugee status are factors that affect the health entitlement of refugees to publicly funded health insurance. Minors (children and young people under the age of eighteen) and refugees fully covered, whereas asylum-seekers, during their first 18 months in Germany have restricted access (*§4 and § 6 Asylum-Seekers’ Benefits Act (AsylBLG))* [32], that in turn complicate assess and delay integration into the German health system. This result reflects findings from Australia [58], and Germany [73,74]. These show that eligibility criteria based on refugee status not only complicated access to health care but also resulted in delayed care, affect treatment outcomes, and increase expenditures.

This research identified access and follow-up issues of some participants related to professional inadequacies. This includes perceived failure to competently convince, agree on, and accommodate patients’ treatment demands. A similar pattern of results was obtained in a systematic review compiled by Keboa and colleagues [28]. As indicated by Hobbs [58], the evidence that we found confirmed that a poor relationship between participants and providers acts as a barrier to seeking dental care. Furthermore, in line with research conducted in Australia [30], access to dental care was inhibited because participants were found to show less adherence to dental treatment. Many of them were found to be poorly motivated and to be less engaged or involved in their own oral health decision-making, including the choice of alternative treatments.

### 4.1. Study Strengths and Limitations

To back up the credibility of our research, we applied the concept of trustworthiness by Lincoln and Guba (1985) [75] with its four components: Credibility, Dependability, Confirmability, and Transferability. Credibility, which relates to the researchers’ ability accurately to identify and describe the study participants [75], was reached through triangulation (both IDI and FGD), prolonged and persistent observations of participants until thematic saturation, and member checking with three participants to verify and provide feedback for their transcript and interpretation of the findings. Dependability, which refers to the consistency of data with time and conditions [75], was appreciated by auditing the rich and thick data set (transcript) against the recorded audio by another Tigrinya-speaking co-author (T.G.) and experienced supervising researcher (C.B.). Confirmability, which closely refers to the objectivity of the research [75], was realised by reflecting on our own preconceptions, bias, backgrounds, and beliefs and thus ensuring that researchers have not influenced the findings in the process of extracting information or analysing the collected data. Yet, being conscious of all these points, and as one of the principal researchers (YSK) is a registered dentist in Eritrea and a Tigrinya speaker, we had continually to reflect on our roles in the research process and the possibility of influencing the response of participants. We embrace this professional perspective and remain as part of the research but at the same time did justice to the shared experiences of our respondents without evaluating them solely from a medical perspective. Transferability concerns how far it is possible to apply methods and findings to other similar study contexts [75]. It was reached by explicitly describing our sample, participants characteristics, methodology and study setting.

The COVID-19 pandemic and subsequent contact restrictions that were in place in Germany during the data-collection phase (March–May 2020) posed some challenges for the research. Due to that, we had to change the initially planned face-to-face into online interviews. Online communication deters the possibility of using the whole spectrum of non-verbal cues and interferes with building a robust relationship between interviewer and interviewee. We addressed that by employing follow-up and probing questions to keep respondents focused throughout and across all the interviews and discussions. We also transcribed, from the recorded video, all the verbal expressions, facial expressions, and emotional intonations of the respondents.

We highlighted a lack of back-translation of the transcripts from English back to Tigrinya as another limitation of this study. Inter-rater reliability could have been approached had another translator back translated the transcripts prior to analysis. We had insufficient funds for a second translator, however, the translations were reviewed for accuracy by both Tigrinya and English-speaking co-author (T.G.).

### 4.2. Practical Implications

The findings suggest that Eritrean refugees and asylum-seekers are not sufficiently well-informed about their overall oral healthcare, nor do they make enough use of oral healthcare services. Acknowledging refugees’ poor oral health status and limited access globally, it is considered that dental services should be included within primary healthcare, and efforts should be made to provide comprehensive dental screening at the first point of entry into host countries.

With regards to issues related to navigating the health system and misconceptions about oral hygiene, authorities and health workers should develop and deliver oral health education, promotion, and outreach activities, to improve awareness, utilisation, and accessibility of dental services. In addition, accessible and understandable information should be provided on scientifically supported oral hygiene measures, preventative dental care, and how to access the German health system. Oral healthcare providers should also build trust with their clients by cultivating a friendly patient-provider relationship, understanding culturally sensitive information, and demonstrating intercultural competency.

Enhancing oral health literacy in the community, and appropriate dental public health strategies would probably also benefit oral health care among refugees and asylum-seekers. Policy-makers should re-define the current framework (*AsylBLG*) of eligibility criteria for asylum seekers to access dental care only for emergencies, or painful and acute conditions, and that in event involves lengthy administration procedures [32].

Finally, as oral health care of refugees is still a neglected field, we recommend further research that focuses on oral healthcare professionals’ experience and perspective of dental care services.

## 5. Conclusions

Eritrean refugees and asylum seekers have a fairly realistic perception and understanding of oral health. However, the majority have poor dental care behaviour or practice, whilst a few have certain misconceptions of the conventional oral hygiene tools. This study uncovered that the majority of the participants were mainly concerned about the psychosocial attributes of poor oral health rather than its functional implications. Since their arrival in Germany, the participants have been found to be influenced by the global dietary transition. This is not only a contributing factor to the rising burdens of non-communicable diseases (NCDs) worldwide [76], but is also believed to be the leading cause of their poor oral health. This study has shed light on participants’ reported barriers to oral healthcare services. Along with the individual’s or client’s own ability barriers, communication barriers remain the main hurdle at all stages of accessing oral healthcare. That includes problems with identifying and navigating oral health services. Additionally, it interferes with initiation or building up patient-doctor relationships. It further interferes with what to do when patients arrive at the dental surgery, how to decide on payment arrangements, and whom patients should trust. To address the oral health burdens of ERNRAS, it is prudent to consider language-specific, inclusive, and culturally and professionally appropriate oral health care services. Only then the Universal Health Coverage (UHC) of the Sustainable Developmental Goals 3 (SDG 3) could be achieved.

## Figures and Tables

**Table 1:** Socio-demographic characteristics of participants.

		*n*	
Age range (Years)			
	18–25	11	44%
	26–35	9	36%
	36–45	5	16%
	46–55	1	4%
Gender			
	Male	19	76%
	Female	6	24%
Educational Level (Years attending school)			
	Primary (1–5)	2	8%
	Middle (6–8)	4	16%
	Secondary (9–12)	14	56%
	Higher (13+)	5	20%
Marital Status			
	Married	7	28%
	Unmarried	18	72%
Employment Status			
	Employed	15	60%
	Unemployed	10	40%
Stay in Germany (Years)			
	≤3	7	28%
	>3	19	72%
Place of Residence			
	Heidelberg	15	60%
	Eppelheim	3	12%
	Plankstadt	2	8%
	Dossennheim	3	12%
	Bammental	2	8%
Refugee Status			
	Refugee	21	84%
	Asylum seeker	4	16%

## Data Availability

The data presented in this study are available on request from the corresponding author.

## Appendix A

**Table A1:** Wide-ranging questions of the IDI and FGD.

No.	
1.	What is the first thing that comes to your mind when you hear about oral health?
2.	What is good oral health to you? What about oral healthcare?
3.	How concerned are you about your oral health?
4.	What is your opinion on the relationship of poor oral health and general health?
5.	Thinking as ERNRAS, how would you describe your overall oral health status?
6.	What do you think the risk factors for the poor oral health among ERNRAS?
7.	Can you talk about the oral hygiene tools you are using? How often are you using?
8.	what is your opinion on ‘when a dentist should be visited?’
10.	Can you tell me what are the main factors hindering ERNRAS from demanding oral healthcare services in Germany?
11.	what is your opinion on how oral health issues of refugees should be managed at the individual, community, governmental or policy levels?

## Appendix B

**Table A2:** Themes, sub-themes, and exemplar quotes.

Themes and Sub-themes	Pertinent Findings According to Participants (ERNRAS)	Quotes
**Perception of Oral Healthcare**		
Perceived definition of oral health	Oral health is related to possessing of white teeth, pleasing smile with no broken, decayed, or crowded tooth and also no bleeding gums or oral malodour.	*“we shouldn’t have a dental cavity, oral ulcers, bad mouth odour or no broken or crooked tooth” (FGD-1).*
Social acceptance and Self-esteem	Sound and satisfactory oral health status believed to enhances self-esteem and social approval, and opposite is true in case of poor oral health. Routine consumption of a traditionally prepared spice blended pepper (Berbere), is believed to prevent bad breath and its burdens.	*“If we don’t have good teeth, we don’t have a girlfriend (laughter)” (FGD-1).* *“After I knew from my friends about the smell of my mouth […], it wasn’t good news […], I soon lost my confidence and couldn’t stand talking to people.” (IDI-14).* *“Then, I wouldn’t stop eating Berbere so that I could stay free of bad breath” (IDI-8).*
**Understanding of Oral Health Determinants**		
Awareness of oral health as holistic health	They acknowledged the close relationship between oral health and the overall health	*“Tooth has to be cleaned and kept healthy so that we can eat nutritious foods and we live longer” (IDI-11).*
Perceived risks of poor oral health	lifestyle change and dietary transition from low-sugar food in Eritrea to high-sugar content in Germany was believed as the main cause of their current poor oral health conditions.	*“Here [Germany], we, Eritreans, are consuming a lot of sweet, soft, and packed food, unlike the food we used to eat in our country, which was hard to chew and less sweet. I believe this is the reason for this poor oral health” (IDI-5).*
**Dental Care Behaviour**		
Personal dental care	All practicing some form of oral hygiene habits that varies from, once or twice a day to an irregular basis using toothbrush, paste, mouth wash or twig (Mewets). They either not using or unaware of dental floss. A shift from the habit of using twig (Mewets) to toothbrush and is believed to be due to unavailability of the right tree in Germany.	*“I always clean my teeth in the morning, right after I eat my breakfast, and sometimes in the evening—before I went to bed” (IDI-3).* *“I don’t have any idea about this thread, and I have never used one in my life” (FGD-2).* *“Not always and only, but I sometimes use Mewets if I can find a good tree” (IDI-15).*
Professional dental care	The Majority attend dental clinic in times of dental emergency or pain. Only 2 (8%) regularly (twice a year), another 2 (8%) never attended, and 17 (68%) at least once in their lifetime.	*“I only go to a dentist for an essential treatment; for example, I once went to a dental clinic for a severe dental pain” (IDI-3).* *“I check my tooth every six months; it doesn’t matter whether I have a problem or not.” (IDI- 2)*
Misperception of oral healthcare practice	Some believed that continuous use of toothbrush and paste, could harm their teeth, a reason for introducing and widening spacing between teeth, and intermittent use of toothbrush as a risk factor for bad breath. Flossing perceived to damage gums and initiation of flossing addiction that in turn exacerbates the negative effect of flossing.	*“From my understanding […], and from what I heard, the chemical in the toothpaste is destructive to our teeth.” (FGD-1).* *“I guess it might damage our teeth or gum. So, I don’t have any plans to use it” (IDI-14)*
**Approachability and Ability to Perceive**		
Information about availability and navigation of the oral healthcare system	ERNRAS lack clear information to locate dental and navigate the oral healthcare system in Germany. They lack information on eligibility (service fee exemption), dental care entitlement and health insurance.	*“I don’t know where and how to find it [dental clinic” (IDI-6).* *“I don’t know even whether a regular visiting is free. I am just hearing now that I could go to a dentist on a twice a year basis”(IDI-12).*
Oral health literacy and beliefs	Few lacks clear understanding regarding the vital significance of oral health care. Awareness issues while they were in Eritrea influenced the poor oral healthcare outcome and mentality in Germany. They have beliefs in homeopathic medicines such as potions, herbs, and saltwater rinse, praying, tolerating, or fighting dental pain affected their perception.	*“In our case [Eritreans], […] we were neither screened nor taught to take care of our teeth when we were in our country. Then we grew up known nothing, and it is costing us a lot to learn to take good care of our teeth. We are simply detached of the reality” (FGD-2).*
The level of trust and Expectations from a dentist	Perceived negatively for some of their dentists’ treatment decisions, the bureaucracy of the dental clinic, and worried about future financial implications.	*“When they [dentists] are taking out our tooth, they want to use it [tooth] for their interest and replace ours with artificial tooth”(FGD-1).*
**Acceptability and Ability to Seek**		
Interculturally competent professionals	Some dental care professionals fail to consider the uniqueness of ERNRAS regarding their cultural views, practices, and beliefs towards oral healthcare acts as a barrier to access care.	*“I can say my first dentist could have done more […]. Not only he doesn’t want to hear my opinion, but also wanted me to follow his instructions only. That’s why the treatment he provided that time couldn’t satisfy me” (IDI-3).*
Lack of communication and professional value	Barriers related to dental care professionals’ failure to communicate patiently or productively, and also poor professional conduct and racial discrimination.	*“There is one staff, she doesn’t really hear you what you want to say, I don’t know why, she is either racist or arrogant” (IDI-4).*
Autonomy and capacity to seek oral healthcare	ERNRAS were unable to seek dental care due to their limited capacity and doubtful self-reliance related to language barrier.	*“Sometimes, even though we are in a great misery and needed treatment, we don’t go to the dentist due to lack of confidence the language barrier puts us into” (FGD-1).*
Disregard or negligence	Participants fail to seek dental care and regular check-ups related to their negligence, disregard, and unfavourable previous dental experience.	*“Dental care never been my priority […], unless I have a serious pain, I don’t care to visit a dentist for a minor discomfort” (IDI-6).*
Fear, anxiety and past dental experience	ERNRAS fail to seek or discontinue dental treatment due to apprehension related to physical pain, anxiety, and past personal or friends’ negative dental experience.	*“Dental appointment is good, [..] but I would never go to my dentist for a regular check-up unless I have pain; I am scared of the machines”(FGD-2).*
**Availability and Accommodation and ability to reach**		
Language issues and Availability of translator	Communication attributes hampers access to dental care to ERNRAS: language difficulty, unavailability of translator, and interpreter dependency and privacy concerns.	*“It is the language problem I have; I can’t tell a dentist what is really happening to me, and that is why I didn’t go to the dentist” (IDI-6).*
Existence of dental services, hours of opening and appointment	Access was hindered by delay in appointment, long waiting list, inconvenient or inflexible clinic working hours.	*“Sometimes the long waiting time and all […], we [refugees] don’t visit unless we have serious problem or pain” (IDI-11).*
Living environment and mobility	Eritrean refugees, particularly those living in the small towns or villages, raised the impact of long distance in reaching dental clinic.	*“Well, earlier my dental clinic wasn’t that far, now that I have changed my residence area to small village, it’s a bit far from my dental clinic. I am not going to the dental clinic, maybe because of this” (IDI-5).*
**Affordability and Ability to pay**		
Direct and Indirect cost	Unaffordability of direct payments (out-of-pocket, co-payment) and indirect costs (transport, dental product, and opportunity cost) were raised as barriers by most of the participants.	*“And, the other day that I didn’t visit my dentist might have been due to the instinct I developed to avoid paying money to the dentist as it is crazy expensive”(IDI-3).*
Entitlement based on age, refugee and employment status	Adult asylum seekers were unable to access dental care as they were not entitled to (except in cases of emergency). Unemployed (inadequate income) ERNRAS unable to pay dental services that require fee or co-payment and fail to access dental care.	*“I wanted to check and clean my teeth. However, I couldn’t get the treatment, both cleaning and filling my teeth, [..] because I wasn’t entitled to that treatment as I was an asylum seeker” (FGD-2).* *“Dental treatment is so expensive that I can’t really afford it since a I am not working right now” (IDI-3).*
**Appropriateness and ability to engage**		
Adequacy and quality of the dental care providers	ERNRAS claimed that their accessibility to dental care was also hampered by inadequacy and incompetence of the oral healthcare providers.	*“I always think that, at my first visit, the dentist could have given me more information. He [dentist] should have checked my teeth very well and he should have filled it instead of removing” (IDI-3).*
Provider-patient relationships	Poor relations with the dental team discourage ERNRAS from accessing dental care.	*“Some of the nurses in the reception shows you bad attitude and are not friendly” (IDI-2).*
Adherence and involvement of ERNRAS in dental treatments	Participants’ fail to engage in decision-making and adherence to the recommended dental care. ERNRAS were inadequately motivated, committed, and less involved in their comprehensive oral healthcare decision-making.	*“Most of us [refugees] are not registered with the town’s refugee collaboration community, where we could have participated and seek help when we face difficulty in understanding and accessing the dental services” (IDI-7).*
